# A Threshold-Based Max-log-MPA Low Complexity Multiuser Detection Algorithm

**DOI:** 10.3390/s20041016

**Published:** 2020-02-13

**Authors:** Guanghua Zhang, Zonglin Gu, Qiannan Zhao, Jingqiu Ren, Weidang Lu

**Affiliations:** 1 School of Electrical Engineering and Information, Northeast Petroleum University, Daqing 163318, China; dqzgh@nepu.edu.cn (G.Z.); guzonglingzl@163.com (Z.G.); zhaoqiannan824@163.com (Q.Z.); 2 College of Information Engineering, Zhejiang University of Technology, Hangzhou 310014, China; luweid@zjut.edu.cn

**Keywords:** sparse code multiple access, threshold, message passing algorithm, multiuser detection, maximum likelihood logarithm

## Abstract

Sparse Code Multiple Access (SCMA) technology is a new multiple access scheme based on non-orthogonal spread spectrum technology, which was proposed by Huawei in 2014. In the algorithm application of this technology, the original Message Passing Algorithm (MPA) has slow convergence speed and high algorithm complexity. The threshold-based MPA has a high Bit Error Ratio (BER) when the threshold is low. In the Maximum logarithm Message Passing Algorithm (Max-log-MPA), the approximation method is used, which will cause some messages to be lost and the detection performance to be poor. Therefore, in order to solve the above problems, a Threshold-Based Max-log-MPA (T-Max-log-MPA) low complexity multiuser detection algorithm is proposed in this paper. The Maximum logarithm (Max-log) algorithm is combined with threshold setting, and the stability of user nodes is considered as a necessary condition for decision in the algorithm. Before message updating, the user information nodes are judged whether the necessary conditions for the stability of the user node have been met, and then the threshold is determined. Only users who meet the threshold condition and pass the necessary condition of user node stability can be decoded in advance. In the whole process, the logarithm domain MPA algorithm is used to convert an exp operation and a multiplication operation into a maximum value and addition operation. The simulation results show that the proposed algorithm can effectively reduce the computational complexity while ensuring the BER, and with the increase of signal-to-noise ratio, the effect of the Computational Complexity Reduction Ratio (CCRR) is more obvious.

## 1. Introduction

In recent years, with the globalization, informatization and the coming of network era, the number of mobile communication users has increased explosively [1], and the demand for network in different places is also increasing rapidly. Mobile networks need to have large-scale connection, super density, wide coverage, high capacity, and low delay to meet these needs [2,3,4]. But in the large-scale access scenario of the Internet of Things, because each resource block in the Orthogonal Multiple Access (OMA) technology [5] is allowed to be allocated to only one user, the number of users allowed access is greatly limited. At the same time, OMA technology is unable to meet the needs of the new generation of mobile communication systems due to the limited spectrum resources [6]. The new 5G air interface technology has the characteristics of fast speed, wide range, and unlimited continuity, and has high flexibility and adaptability with its own core technology and advantages [7]. In addition, 5G communication technology also has 25 times the throughput and more than 10 times the resource utilization of 4G [8,9]. Sparse Code Multiple Access (SCMA) is one of the 5G candidate technologies, and it is the extension and promotion of the Low Density Spread Spectrum (LDS) [10,11]. In the SCMA system, the process of Quadrature Amplitude Modulation (QAM) [12] mapping and the spread spectrum are integrated to form a SCMA codebook [13,14], which brings shape gain to the SCMA coding process [15,16]. SCMA technology is a popular Non-Orthogonal Multiple Access (NOMA) form of multiuser detection using a classical Message Passing Algorithm (MPA) [17], but it has high complexity, so many domestic scholars have conducted in-depth research on reducing the complexity detection algorithm [18,19]. In reference [20], a threshold based MPA is proposed on the basis of codeword reliability. The algorithm calculates the codeword reliability in the iterative process. When the codeword reliability reaches the threshold condition, the user will be decoded in advance, and no message update will be carried out in the subsequent process, so as to reduce the complexity of multiuser detection. However, it will cause the loss of the posteriori soft information of other users who occupy the same resource block with the user in the subsequent iterative process, and also lead to the likelihood operation. With the decrease of precision, the Bit Error Ratio (BER) performance of users will be reduced, especially in the case of a low threshold. In reference [21], the Maximum logarithm Message Passing Algorithm (Max-log-MPA) in the logarithmic domain is used to convert exponential (exp) operation and multiplication operation into maximum value and addition operation [22,23], which reduces the operation complexity. However, the algorithm uses approximate calculation when calculating the message update of resource nodes, resulting in the loss of some information and the degradation of its detection performance.

In order to solve the problem of poor detection performance and the loss of soft information in the iterative process of the original MPA, a new algorithm combining the maximum logarithm and threshold setting is adopted in the paper, and the stability decision of user nodes is also added to the algorithm. Before updating the message, the stability of the user information nodes and whether it has passed the threshold value are studied first, and then the exp operation and multiplication operation are transformed into the maximum value and addition operation by the Max-log-MPA algorithm in the logarithmic domain, which not only improves the detection performance, it can also effectively change the comparison of the complexity reduction rate of the detection algorithm.

## 2. SCMA System Model

The transmitter of the SCMA system consists of multidimensional modulation and sparse spread spectrum [24,25]. The physical layer receiver of the SCMA system consists of a channel equalizer, a multiuser detector, and an error correction decoder. A channel equalizer mainly eliminates the inter-symbol interference caused by channel fading and multipath effects. The main function of a multiuser detector is to distinguish user information loaded on K codebooks and remove the interference between user information. The main function of an error-correcting decoder is to decode each layer of user information and get the received signal.

**Figure 1:**
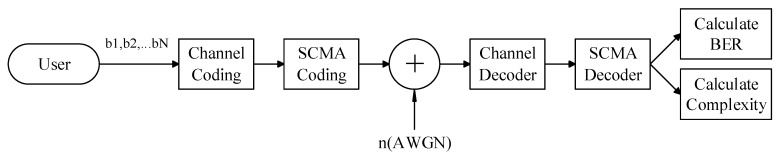
Uplink Sparse Code Multiple Access (SCMA) system block diagram.

Supposing a SCMA multiuser communication uplink system, where j users share k orthogonal resource blocks and transmit through additive white Gaussian noise, the specific process is shown in Figure 1 below. After user information data $u_{j}(j\in [1,2,\cdots ,J])$ is coded by a forward error correction channel [26], the codeword set $x_{j}$ corresponding to user j sends a bit stream $b_{j}$ to the SCMA encoder and maps it to the K-dimensional resource node. Each user has a different codebook. The codebook of the j-th user is $x_{j}$, and $b_{j}$ represents the information bit data of the j user. Let the received signal on the K orthogonal resource blocks be $y={\left[y_{1},y_{2},\cdots ,y_{K}\right]}^{T}$, then the user information is transmitted through the channel and the received signal is:

(1) $$y=\sum_{j=1}^{J}\mathrm{diag}(h_{j})x_{j}+n$$

Among them, $h_{j}={\left[h_{1},h_{2},\cdots ,h_{K}\right]}^{T}$ denotes the channel fading coefficient vector. $x_{j}={\left[x_{1,j},x_{2,j},\cdots ,x_{K,j}\right]}^{T}$ represents the SCMA code character number of the j-th user.$\mathrm{diag}(h_{j})$ means diagonal matrix.$n(n(0∼N_{o}{}^{2}))$, which denotes the additive white Gaussian noise vector [27].

M is the number of codewords in the codebook, that is, the size of the codebook. M depends on the number of bits of binary data. User information data is divided into several groups according to the a-bit group, and a-bit user information data is the size of codebook:

(2) $$M=2^{a}$$

Because of the sparsity of SCMA, when the transmitter of SCMA maps the log_2_M bit data to a sparse codeword symbol in the k-dimension codebook, the mapping relationship can be expressed as follows:

(3) $$f:B^{\log_{2}M}\to \chi ,x=f(b_{j})$$

Among them,χ is the user’s codebook, its dimension is K, K is the spread spectrum factor, B is the set of binary numbers, and the SCMA codebook in the user’s j codebook is $x_{j}$. Then, the process of user binary data $b_{j}$ coding to the SCMA codeword is as follows:

(4) $$x_{i}=f(b_{i})$$

Suppose that each user occupies an average of N(1≤N≤K) resources in the SCMA system. In order to distinguish different users, there is at least one difference between resource blocks occupied by any two users, and the maximum number of users carried on each resource block is:

(5) $$J_{\max}=\left(\begin{array}{l} K\\ N \end{array}\right)=C_{N}^{K}=\frac{K(K-1)\cdots (K+1-N)}{N(N-1)\cdots 1}$$

The actual number of users per resource block is:

(6) $$M=\left(\begin{array}{l} K-1\\ N-1 \end{array}\right)=C_{N-1}^{K-1}=\frac{\left(K-1)(K-2)\cdots (K+1-N\right)}{(N-1)(N-2)\cdots 1}=\frac{JN}{K}$$

The ratio representing the number of users that users can bear on a certain resource block is called the overload factor, and its calculation method can be expressed as:

(7) $$\lambda =\frac{J}{K}=\frac{\left(K-1)(K-2)\cdots (K+1-N\right)}{N(N-1)\cdots 1}(J\geq K)$$

Formula (7) shows that when λ>1, K≥4(2≤N≤K−2), the system can achieve the purpose of overloading at this time. When N=1, K=J, the SCMA system is equivalent to the traditional Orthogonal Frequency Division Multiple Access (OFDMA) system. Therefore, OFDMA is a special case of SCMA. In OFDMA, when λ=1 the system achieves full load, that is, an overload condition of SCMA. In order to maintain the sparsity of the system, 2 ≤ N ≤ K/2 is usually chosen. In particular, when N = 2, the system has the strongest sparsity. So, the case of N = 2 is focused in the paper.

**Figure 2:**
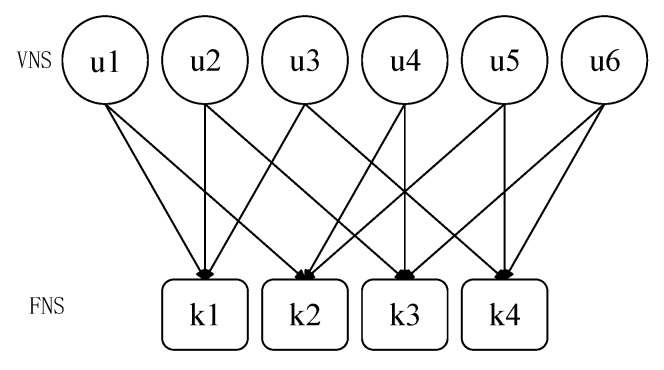
Tanner diagram of codebook (J = 6, k = 4).

Suppose there are 6 users and 4 time-frequency resource blocks, that is, the overload rate is 150%. The relationship between them can be represented by a Tanner diagram, as shown in Figure 2. That is to say, user information $x_{j}$ is the process of sending from the variable node (VN) to the function node (FN).

**Figure 3:**
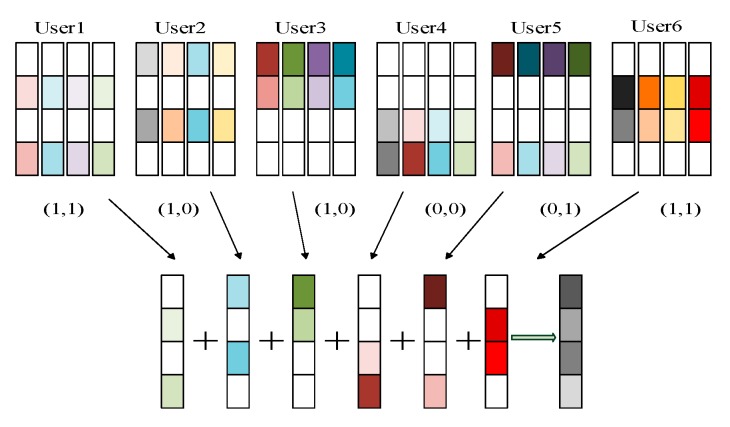
SCMA coding principle.

At this time, six users send signals at the same time, and the information bits from user 1 to user 6 are respectively sent as shown in Figure 3 [28]. 

The codebook corresponding to each user is superimposed on the four time-frequency resource blocks used. Each user has a unique codebook, and each codebook is a 4×4 complex matrix. Each user’s codebook has four codewords. After channel coding from left to right, the selected binary bit string 0-1 represents the user’s transmission on the time-frequency resource block. In Figure 2 and Figure 3, the position of non-zero elements in user 1 is 2, 4, which means that the user only transmits signals on orthogonal time-frequency resource 2 and orthogonal time-frequency resource 4. Similarly, other users’ information transmission can be known. In order to express this transmission more conveniently, the sparse matrix $F_{K\times J}$ is introduced, and the sparse matrix in Figure 3 is as Formula (8):

(8) $$F_{4\times 6}=\left[\begin{array}{cccccc} 0 & 1 & 1 & 0 & 1 & 0\\ 1 & 0 & 1 & 0 & 0 & 1\\ 0 & 1 & 0 & 1 & 0 & 1\\ 1 & 0 & 0 & 1 & 1 & 0 \end{array}\right]$$

In the formula, the rows in the $F_{K\times J}$ matrix represent resource blocks and the columns represent user codewords. 0 in the matrix represents that the user does not transmit the signal in the corresponding time-frequency resource block, and 1 represents the transmission signal.

## 3. Original MPA Algorithm

A single complex problem is decomposed into several simple problems in MPA, and based on the joint posterior probability, the loss of information in the process of factor transfer is reduced as much as possible. The sparse property of the factor graph is used to iteratively update and transfer between variable nodes (VN) and functional nodes (FN) in MPA [29]. Assuming that the codewords sent by each user are of equal probability distribution, the first step is to initialize the posteriori probability of each codeword of each user and calculate the conditional probability:

(9) $$I_{c_{k}\to u_{j}}^{0}(x_{j})=\frac{1}{M}$$

where $I_{c_{k}\to u_{j}}^{t}(x_{j})$ represents the message from the resource node to the user node. k (k = 1, 2, …) denotes the ordinal number of the resource nodes. $c_{k}$ denotes the k-th resource node, j (j = 1, 2, …) denotes the user node number, and $u_{j}$ denotes the i-th user node.

The second step is to update the information value from the resource node to the user node. The result of initialization calculation is used to carry out multiple transmission iterations of information. The two stages in the process of one iteration can be expressed by mathematical formulas as follows:

(10) $$I_{c_{k}\to u_{j}}^{t}(x_{j})=\sum_{∼x_{j}}\left\{\frac{1}{\sqrt{2\pi \delta }}\exp{\left(-\frac{1}{2\delta^{2}}\left\|y_{k}-\sum_{v\in \xi_{k}}h_{k,v}x_{k,v}\right\|\right)}^{2}\times \prod_{m\in \xi_{k}/j}I_{c_{m}\to u_{k}}^{t}(x_{j})\right\}$$

(11) $$I_{u_{j}\to c_{k}}^{t}(x_{j})=\prod_{m\in \xi_{k}/k}I_{c_{m}\to u_{j}}^{t}(x_{j})$$

where t denotes the number of iterations, $\xi_{k}$ and $\xi_{j}$ represent the set of non-zero elements of row k and column j in the sparse code matrix $F_{4\times 6}$. $x_{v,k}$ denotes the codeword of the v-th user on the k-th resource block. $h_{k,v}$ denotes the channel coefficients of the v-th user on the k-th resource block.

In the third step, after passing the maximum number of iterations previously set, the decision output is performed. The output probability of the code character number decoded by MPA is as follows:

(12) $$Q(x_{j})=\prod_{k\in \xi_{j}}I_{c_{k}\to u_{j}}^{t_{\max}}(x_{j})$$

**Figure 4:**
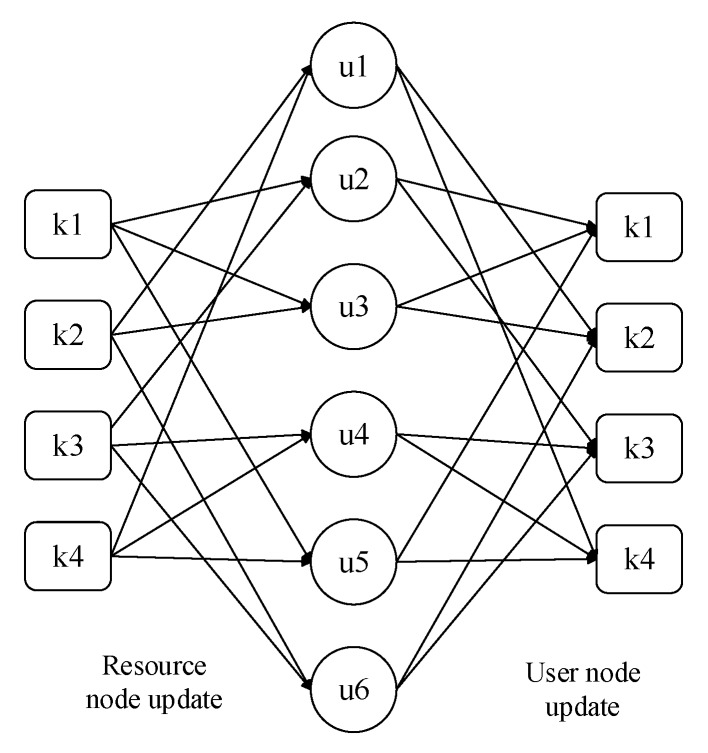
**The** Update process of MPA algorithm

There are two steps in each update process: the update of the resource node and the update of user node, as shown in Figure 4.

## 4. Threshold-Based MPA

In threshold-based MPA, each user node is updated iteratively. When the minimum number of iterations t_min_ is reached, the Likelihood Rate (LLR) of the user coding bit can be judged whether it meets the threshold requirement. If it meets the requirement of setting the user flag bit from 1 to 0, it means that the user will stop iteratively updating after that. Because, when the maximum likelihood ratio of the codeword reaches the threshold requirement, the user can judge the transmitted codeword more accurately and stop updating the user in the subsequent iteration process. The method reduces the number of user nodes that need to be updated in each cycle, thus reducing the complexity of the system.

(13) $${\mathrm{LLR}}_{j,x}=\log(\frac{P(b_{i}=0)}{P(b_{i}=1)})=\log(\frac{\sum_{m:b_{m,i}=0}Q(x_{j,m})}{\sum_{m:b_{m,i}=1}Q(x_{j,m})})$$

where ${\mathrm{LLR}}_{j,k}$ denotes the log LLR. $P(b_{i}=0)$ denotes the probability of decoded user nodes. $P(b_{i}=1)$ denotes the probability of waiting for decoding user nodes.

Define th is the setting threshold value. Assuming LLE_exp=exp(LLR), the required threshold is: if the first bit of user j satisfies the threshold $\mathrm{th}\leq \mathrm{LLE}\_\exp_{j,1}<1/\mathrm{th}$, the user information is determined as 0, no more iteration update. If the first bit of user j does not meet the threshold requirement $\mathrm{LLE}\_\exp_{j,1}<\mathrm{th}$ and $\mathrm{LLE}\_\exp_{j,1}\geq 1/\mathrm{th}$, the user information decision is 1, continue with iteration update. If the second bit of user j satisfies the threshold $\mathrm{th}\leq \mathrm{LLE}\_\exp_{j,2}<1/\mathrm{th}$, the user information is determined as 0, stop iteration update. If the second bit of user j does not meet the threshold requirement $\mathrm{LLE}\_\exp_{j,2}<\mathrm{th}$ and $\mathrm{LLE}\_\exp_{j,2}\geq 1/\mathrm{th}$, the user information decision is 1, continue with iteration update cycle. 

If the threshold requirement, the number of cycles, and the flag bit requirement are met, the transmission codeword ${\mathrm{Codenum}}_{j}$ of user J can be expressed as:

(14) $${\mathrm{Codenum}}_{j}=2\times (\mathrm{LLE}\_\exp_{j,1})+(\mathrm{LLE}\_\exp_{j,2})+1$$

## 5. Threshold-Based Max-log-MPA Algorithm

Because the original MPA requires all codewords to reach the maximum number of iterations t_max_ before the decision can be made, the complexity is very large. Moreover, the exp algorithm takes up a lot of computation and memory space, which results in a high complexity of original MPA [30]. Although the threshold-based MPA algorithm can reduce the complexity of the system, it needs to decode the user whose codeword reliability meets the threshold condition in advance, which will cause the loss of the posteriori soft information of other users who occupy the same resource block with the user in the subsequent iteration process, and also lead to the reduction of the likelihood operation accuracy, and ultimately reduce the performance of the user’s bit error rate. The performance degradation of this bit error rate is especially prominent when the threshold is low. Finally, the BER performance of users is reduced, especially when the threshold is low. The Max-log-MPA algorithm [31], although it can effectively reduce the computational complexity of detection, will make the system BER performance decline. In order to make up for these shortcomings and reduce the complexity of detection, a Threshold-Based Max-log-MPA (T-Max-log-MPA) algorithm detection algorithm is proposed in the paper.

In this paper, codeword reliability and user node stability are combined to measure the reliability of the user codeword. Based on the threshold-based MPA algorithm, the algorithm proposed in the paper adds the judgment of the necessary conditions for the stability of user nodes. Before updating the message, it judges whether the user information nodes meet the necessary conditions for the stability of user nodes, and then it judges whether they pass the threshold conditions. Only users who meet the threshold conditions and pass the necessary condition of user node stability can be decoded in advance. In this way, not only the reliability of the early decision codewords are improved but the loss of the posteriori soft information caused by the detection mechanism of the changed phase hard decision is also reduced. Especially in the case of a low threshold, the message can be iterated more fully, which improves the BER performance of a threshold-based MPA. In addition, by taking the advantages of the Max-log-MPA algorithm, the logarithmic domain algorithm is used to transform exp operation and multiplication operation into a maximum value and addition operation in the whole iterative updating process, which can not only effectively reduce the complexity of operation, but also keep good BER performance.

The “user node stability” used in the paper refers to the SCMA iterative updating process, if the position of the largest element in the codeword credibility vector of user node $u_{j}$ in the factor graph in the i-th iteration is the same as that in the $i^{\prime }$-th iteration. That is, $\underset{1\leq m\leq M}{\mathrm{argmax}}\;q^{i}\left(\chi_{j,m}\right)=$ $\underset{1\leq m\leq M}{\mathrm{argmax}}\;q^{i^{‘}}\left(\chi_{j,m}\right)$, $i<i^{\prime }\leq T_{\max}$, of the i and i+1 iterations. It is also equivalent to m = n, which indicates that the user node $u_{j}$ is stable. Therefore, the necessary condition for the stability of the user node can be expressed as the position of the largest element in the codeword credibility vector, which is the same in the i-th and i+1-th iterations, that is, $\underset{1\leq m\leq M}{\mathrm{argmax}}\;q^{i}\left(\chi_{j,m}\right)=$ $\underset{1\leq n\leq M}{\mathrm{argmax}}\;q^{i+1}\left(\chi_{j,n}\right)$ in the i-th and i+1-th iterations is also equivalent to m = n.

For resource node updating user information, the Jacobi algorithm formula is used in this paper, as shown in Formula (15).

(15) $$\log(\sum_{i=1}^{N}\exp(f_{i}))\approx \underset{i=1,2,\cdots ,N}{\max}\left\{f_{1},f_{2},\cdots ,f_{N}\right\}$$

In initialization, assuming that all user nodes are unstable, the iteration process is divided into two parts: message update part $\phi_{i}$ and message validation part $\varphi_{i}$. The message updating parts are shown in Formulas (16)–(18). After the message update, a message validation step is used to determine whether the codeword can be judged ahead of time. The message validation stage is divided into the following steps: the first step is to determine whether the stability of the user node in the untrusted set $\varphi_{i}$ meets the necessary condition of the stability of the user node. If it is reached, the untrusted set $\varphi_{i}$ is directly transferred out and stored in the trusted set $\phi_{i}$ for threshold judgment. The second step is to determine whether the message node in the trusted set $\phi_{i}$ of the user node has passed the decision of threshold value. User nodes that fail to pass the threshold decision are stored in the untrusted set $\varphi_{i}$ and the message update iteration is restarted. In the third step, if the user message nodes not only pass the necessary conditions for the stability of the user nodes but also pass the threshold decision, they are decoded and eliminated in advance, and they are no longer involved in the subsequent iteration updates.

The update step of the information value from the resource node to the user node in the original MPA algorithm can be rewritten as follows:

(16) $$I_{c_{k}\to u_{j}}^{t}(x_{j})=\underset{i=1,2,\cdots ,N}{\max}\left\{-\frac{1}{2\delta^{2}}\left\|y_{k}-\sum_{v\in \xi_{k}}h_{k,v}x_{k,v}\right\|+\sum_{v\in \xi_{k}}I_{u_{j}\to c_{k}}^{t-1}(x_{j})\right\}$$

(17) $$I_{u_{j}\to c_{k}}^{t}\left(x_{j}\right)=\sum_{v\in \varepsilon_{k}/m}I_{c_{m}\to u_{j}}^{t}\left(x_{j}\right)$$

After the maximum number of iterations, the probability of the code character number output after MPA decoding is as follows:

(18) $$Q\left(x_{j}\right)=\sum_{v\in \varepsilon_{j}}I_{c_{k}\to u_{j}}^{t_{\max}}\left(x_{j}\right)$$

The algorithm proposed in this paper will be terminated when all user codewords are pre-judged or the maximum number of iterations is reached.

## 6. Complexity Analysis

The complexity of the original message passing algorithm in the SCMA multiuser detection algorithm is mainly due to the exp algorithm’s complexity [32], large space and iteration, and message updating between variable nodes and functional nodes [33,34]. The algorithm proposed in this paper takes advantage of the necessary condition decision and threshold decision of the stability of user nodes. It only needs a few simple decision operations and greatly reduces the number of iteration cycles. In addition, the logarithmic domain algorithm is used to reduce the exp algorithm to the sum algorithm, which effectively reduces the number of operations. Therefore, in order to compare the complexity of the original MPA, threshold-based MPA, Max-log-MPA algorithm [35] and the algorithm proposed in the paper, it is only necessary to compare the operation amount of the message update link of different algorithms in the iterative process. All the algorithms involved in this paper do not break the message update link. The number of multipliers required for the original MPA is:

(19) $$\mathrm{num}=t_{\max}kd_{f}M^{d_{f}}(2d_{f}+1)+t_{\max}Jd_{v}(d_{v}-2)$$

Among them, num is the number of multipliers, $d_{f}$ and $d_{v}$ represent the number of users per resource block and the number of resource blocks occupied by each user, respectively.

From the above formula, it can be seen that the maximum number of iterations and the threshold are the main factors affecting the algorithm in this paper. Because the Additive White Gaussian Noise (AWGN) is random, the influence of noise on complexity is not considered. Similar to threshold-based MPA, because the necessary condition and threshold decision of user node stability are adopted, user nodes can be decoded ahead of time, the maximum number of iterations can be reduced, and the amount of computation in the message update link can be reduced, so the complexity of the system is reduced and the BER performance is effectively improved.

## 7. BER Performance Analysis

The threshold-based MPA algorithm is essentially a disguised hard decision detection mechanism [36], which can cause the loss of posterior soft information to adversely affect the decision of other user nodes. Even the codewords that could be correctly judged would be judged as errors. As a result, the precision of the likelihood operation is reduced, and the BER performance of SCMA system users is reduced, especially at low threshold. The Max-log-MPA algorithm can also make part of the information lost. Although the BER performance of the system is improved compared with the original MPA, it is still higher than the algorithm in this paper. In this algorithm, the necessary stability condition is determined before the threshold value, which can ensure that the maximum element of the codeword confidence vector is located at the same position in the two adjacent iterations of a user, thus reducing the loss of posterior soft information and improving the accuracy of likelihood operation. Then, the user nodes in the trusted set are judged by threshold and decoded in advance. In the next iteration, no iteration update is carried out, which not only reduces the complexity of the system, but also reduces the BER of user information. Especially when the threshold setting is low, the decision of the necessary conditions for the stability of user nodes is more significant for reducing the BER performance of threshold-based MPA. Finally, iterative updating is performed to reduce the loss of information in the Max-log-MPA algorithm and improve the BER performance of the operation.

## 8. Simulation Results and Analysis

In order to verify the good system performance of the threshold-based low-complexity multiuser detection algorithm of Max-log-MPA proposed in this paper, simulation experiments are carried out to compare it with the original MPA algorithm, the threshold-based MPA algorithm, and the Max-log-MPA algorithm. In the simulation, the parameters are set as shown in Table 1. The codebook used is Huawei’s 4-D codebook published in document [37].

### 8.1. BER Performance

**Figure 5:**
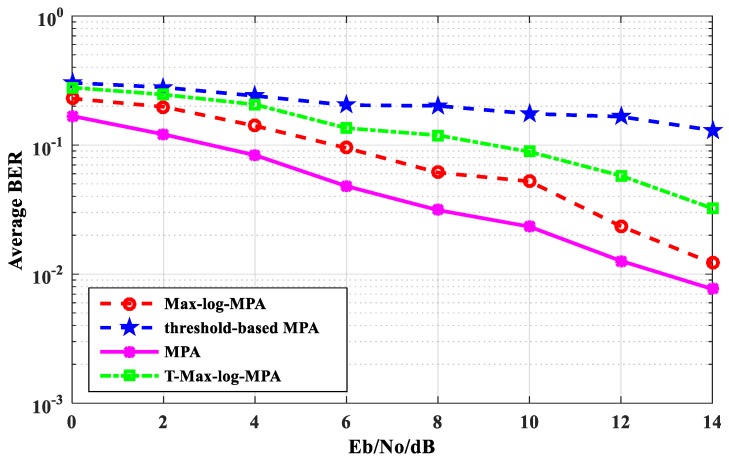
The comparison of average BER performance in four algorithms.

Figure 5 shows the average BER performance comparison of the T-Max-log-MPA algorithm with original MPA, the Max-log-MPA, and the threshold-based MPA. As can be seen from Figure 5, the BER performance of the T-Max-log-MPA algorithm is better than that of the threshold-based MPA algorithm when the maximum number of iterations t_max_ = 5 and the threshold th = 0.60. When E_b_/N_o_ = 14 dB, it is 12.553% lower than the threshold-based MPA algorithm. The BER performance of the T-Max-log-MPA is higher than that of the Max-log-MPA and the original MPA. When E_b_/N_o_ = 0 dB, the BER performance of the T-Max-log-MPA algorithm is 3.86% higher than that of the Max-log-MPA and 11.16% higher than that of the original MPA. When E_b_/N_o_ = 14 dB, it is 0.70% higher than the Max-log-MPA algorithm and 1.767% higher than the original MPA algorithm. According to the comparison results, although the BER performance of the T-Max-log-MPA algorithm is higher than the Max-log-MPA algorithm and the original MPA algorithm, the BER performance of the T-Max-log-MPA algorithm is better with the increase of E_b_/N_o_.

**Figure 6:**
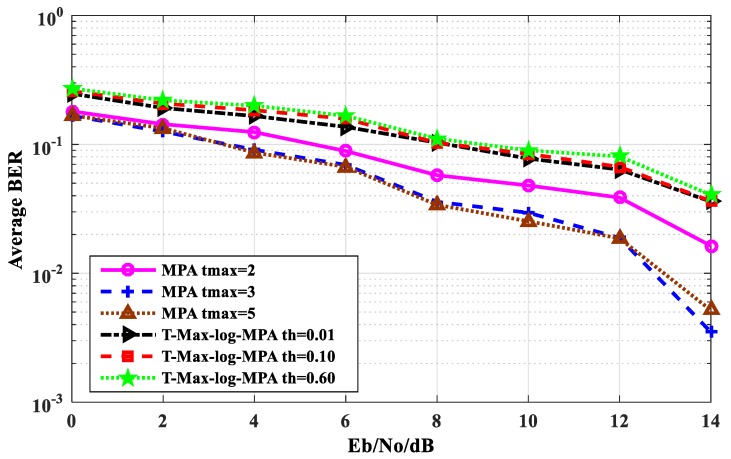
The Comparison of BER performance between the MPA algorithm and the Threshold-Max- log-MPA algorithm.

Figure 6 shows the average BER performance comparison between the T-Max-log-MPA algorithm and the original MPA algorithm. As can be seen from Figure 6, the bigger the threshold of the T-Max-log-MPA algorithm, the less obvious the BER performance change. The BER performance of the threshold th = 0.10 and the threshold th = 0.60 are almost the same. The BER performance of the T-Max-log-MPA algorithm with the threshold value th = 0.01 is the closest to that of the original MPA algorithm t_max_ = 2. When E_b_/N_o_ = 14 dB, the BER performance of the former is 3.633 × 10^−2^, while that of the latter is 1.633 × 10^−^^3^, and the BER performance difference between the two is 2.0 × 10^−2^.

### 8.2. Complexity Comparisons

**Figure 7:**
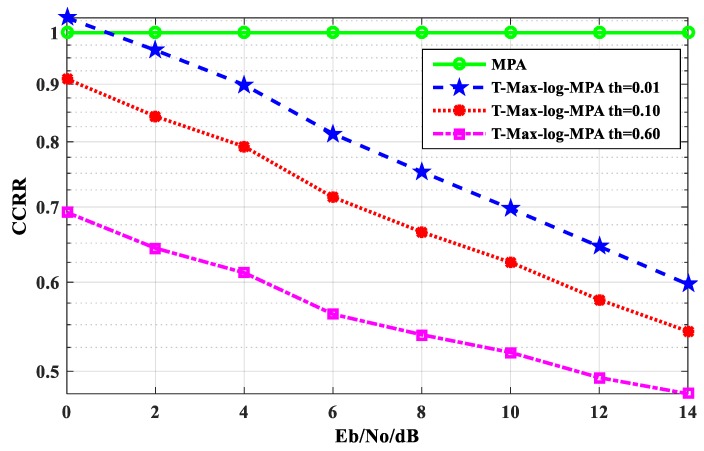
Comparison of the CCRR between the MPA algorithm and the T-Max-log-MPA algorithm.

Data statistics are carried out during the simulation process. In order to compare the complexity of the two algorithms more intuitively, the Computational Complexity Reduction Ratio (CCRR) is used to measure the complexity [38], which is defined as Formula (11). Figure 7 is a comparison of the CCRR of the T-Max-log-MPA algorithm under different E_b_/N_o_, the CCRR of the original MPA algorithm is 1. From Figure 7, it can be seen that the CCRR of the T-Max-log-MPA algorithm, under three different thresholds, is lower than that of original MPA algorithm with iteration times t_max_ = 5. When E_b_/N_o_ =14 dB, the T-Max-log-MPA algorithm is compared with the original MPA algorithm. When the threshold is th = 0.01, CCRR is 38.54% lower than the original MPA algorithm. When the threshold is th = 0.10, CCRR is 44.36% lower than the original MPA algorithm. And when the threshold is th = 0.60, CCRR is 51.21% lower than the original MPA algorithm. Therefore, the CCRR of the system detection algorithm is effectively reduced in the T-Max-log-MPA algorithm. With the increase in the threshold, the reduction effect is more obvious.

(20) $$\mathrm{CCRR}=\frac{\mathrm{Complexity}\;\mathrm{of}\;A\;\mathrm{algorithm}}{\mathrm{Complexity}\;\mathrm{of}\;B\;\mathrm{algorithm}}$$

**Figure 8:**
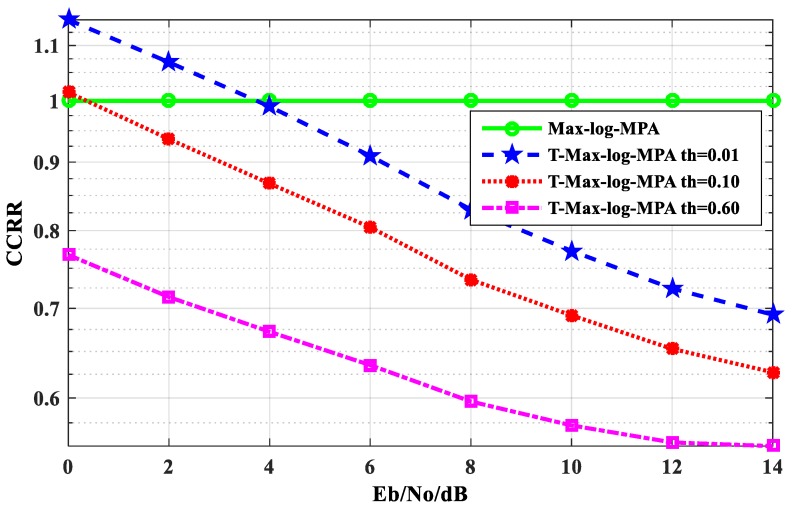
Comparison of the CCRR between the Max-log-MPA algorithm and the T-Max-log-MPA algorithm.

Figure 8 shows the comparison of the CCRR of the T-Max-log-MPA algorithm and the Max-log-MPA algorithm under different E_b_/N_o_ when the maximum number of iterations is t_max_ = 5. It can be seen from Figure 8 that the CCRR of the T-Max-log-MPA algorithm at different thresholds is lower than that of the Max-log-MPA when E_b_/N_o_ > 4 dB. Here, the thresholds is th = 0.01. When E_b_/N_o_ < 4 dB, the T-Max-log-MPA algorithm CCRR is 15% higher than the Max-log-MPA algorithm. When E_b_/N_o_ > 4 dB, the CCRR is lower than the Max-log-MPA algorithm. At E_b_/N_o_ = 14 dB, it is 30.76% lower than the Max-log-MPA algorithm. At the threshold of th = 0.10, the CCRR of the T-Max-log-MPA algorithm is basically lower than that of the Max- log-MPA algorithm. At E_b_/N_o_ < 1 dB, the CCRR is 1.5% higher than the Max-log-MPA algorithm. At E_b_/N_o_ = 14 dB, the CCRR is 37.28% lower than that of the Max- log-MPA algorithm. When the threshold is th=0.60, it is lower than the Max-log-MPA algorithm. At E_b_/N_o_ = 0 dB, the complexity is 23.26% lower than that of the Max- log-MPA algorithm. At E_b_/N_o_ = 14 dB, the CCRR is 44.76% lower than that of the Max-log-MPA algorithm. It can be seen from the figure that the T-Max-log-MPA algorithm can effectively reduce the CCRR of the SCMA system detection algorithm, and with the increase of E_b_/N_o_, the effect is more obvious.

## 9. Conclusions

A T-Max-log-MPA low complexity multiuser detection algorithm for SCMA uplink is proposed based on the research on the traditional algorithm used in this paper. Compared with the original MPA, the algorithm proposed in this paper improves the reliability of the multiuser detection algorithm in determining the codeword in advance, reduces the loss of the posteriori soft information caused by the detection mechanism of the hard decision in the disguised phase, and effectively solves the serious problem of low threshold time error code performance degradation in the threshold-based MPA. By using the logarithm domain algorithm, the exp algorithm and the product algorithm in the original MPA algorithm are transformed into the maximum value and the addition algorithm, which effectively solves the problems of the exp algorithm in the original MPA algorithm, such as a large amount of computation, large memory occupation, high algorithm complexity, and so on. In addition, it can guarantee good BER performance.

## Figures and Tables

**Table 1:** Simulation parameters.

Parameters	Value
Codebook selection	Huawei
Codebook size M	4
Users J	6
Time-frequency resources K	4
Overload factor	150%
Maximum number of iterations $T_{\mathrm{Max}}$	5
Channel model	AWGN
N	1000
Threshold th	0.6
